# Non-ribosomal peptide synthetase (NRPS)-encoding products and their biosynthetic logics in *Fusarium*

**DOI:** 10.1186/s12934-024-02378-1

**Published:** 2024-03-27

**Authors:** Ziwei Huang, Wangjie Zhu, Yifan Bai, Xuelian Bai, Huawei Zhang

**Affiliations:** 1 School of Pharmaceutical Sciences, Zhejiang University of Technology, Hangzhou, 310014 China; 2 College of Life and Environmental Sciences, Hangzhou Normal University, Hangzhou, 310036 China

**Keywords:** *Fusarium*, Secondary metabolite, Non-ribosomal peptide synthetase-encoding product, Biosynthetic gene cluster, Biosynthetic pathway

## Abstract

Fungal non-ribosomal peptide synthetase (NRPS)-encoding products play a paramount role in new drug discovery. *Fusarium*, one of the most common filamentous fungi, is well-known for its biosynthetic potential of NRPS-type compounds with diverse structural motifs and various biological properties. With the continuous improvement and extensive application of bioinformatic tools (e.g., anti-SMASH, NCBI, UniProt), more and more biosynthetic gene clusters (BGCs) of secondary metabolites (SMs) have been identified in *Fusarium* strains. However, the biosynthetic logics of these SMs have not yet been well investigated till now. With the aim to increase our knowledge of the biosynthetic logics of NPRS-encoding products in *Fusarium*, this review firstly provides an overview of research advances in elucidating their biosynthetic pathways.

**Supplementary Information:**

The online version contains supplementary material available at 10.1186/s12934-024-02378-1.

## Introduction

Fungal non-ribosomal peptide synthetases (NRPS) are large modular multifunctional enzymes that generate compounds by sequential condensation of amino acids and hydroxycarboxylic acid units [1]. Fungal NRPS-encoding products are a prolific source of bioactive compounds, some of which have been commercially used as therapeutic agents, such as cyclosporin A, echinocandins and emodepsides [2, 3]. As one of the most common filamentous fungi in nature, *Fusarium* is well-known for its potential of production of NRPS products with a wide array of biological properties [4–6]. With a substantial increase in fungal genome sequences and the incremental optimization of software tools (e.g., anti-SMASH, NCBI, UniProt), bioinformatic analysis of the link between secondary metabolites (SMs) and their biosynthetic gene cluster (BGCs) has become simple and efficient [7–9]. A growing number of *Fusarium*-derived NRPS products and their BGCs have been isolated and characterized [6, 10, 11]. However, the biosynthetic pathways of these SMs have not been well unveiled till now. By extensive literature search and analysis, this review comprehensively summarizes 15 biosynthetic pathways of NRPS-type compounds from *Fusarium* spp., highlighting the key enzymatic domains involved in their biosynthetic pathways. Additionally, the supporting information summarizes some of the common methods, which can provide valid references for further research.

## Canonical NRPS-encoding compounds

One fungal NRPS module usually consists of at least three essential domains including the adenylation (A), the thiolation (T) and the condensation (C) [12–15]. The other family members also can replace the C domain in the biosynthesis or work together with C domain, including the epimerization (E) domain, the heterocyclization (Cy) domain, the CT domain (a subset of the C domain) etc., which can meet diverse and novel functions [16, 17]. The released products are subsequently further modified by additional enzymes, which are encoded by genes located near the NRPS and thus form the final product [18, 19].

### Fusahexin

Fusahexin (**1**), originally derived from strain *F. graminearum* PH-1, represents a cyclic hexapeptide consisting of six amino acid residues and containing an uncommon ether bond between the C-*δ* of proline and the C-*β* of threonine [20, 21]. Phytopathological investigation showed that this substance plays a key role in hyphal growth, attachment, water–air interface penetration and plant infection through regulation of surface hydrophobicity of conidia and the cell wall as well as hydrophobin rodlet formation in *Aspergillus nidulans* [22–25].

**Fig. 1:**
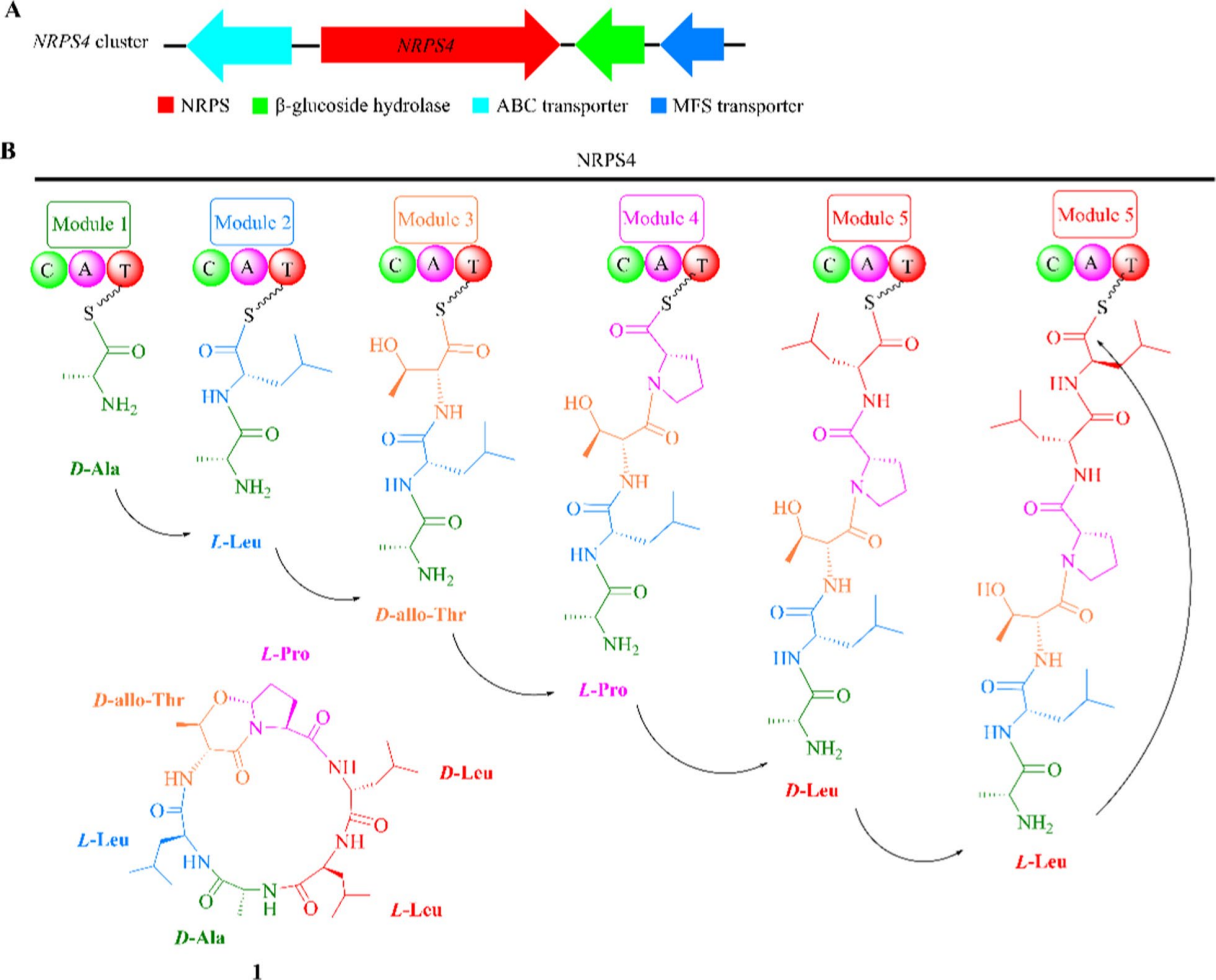
Proposal biosynthetic pathway for fusahexin (**1**). **A** The *NRPS4* gene cluster in *F. graminearum* PH-1; **B** The biosynthetic logic of **1**

Knockout and overexpression experiments revealed that an *NRPS4* cluster in *F. graminearum* was responsible for the production of compound **1** [22, 26]. This cluster contains four genes that respectively encode for glucoside hydrolase, NRPS synthetase (gene *NRPS4*), ABC transporter and major facilitator superfamily (MFS) transporter (Fig. 1A). The NRPS4 enzyme consists of five modules, in which modules 1–4 are respectively responsible for linking *D*-alanine, *L*-leucine, *D*-allo-threonine, and *L*-proline, and module 5 is serially reusable in assembly of *D*-leucine and *L*-leucine (Fig. 1B) [20]. However, the function of other three enzymes in the *NRPS4* cluster had not yet been characterized till now.

### Fusaoctaxin

Fusaoctaxins A (**2**) and B (**3**), two unusual linear and C-terminally reduced octapeptide with *D*-amino acid-rich residues, were novel virulence factors during wheat infection and were firstly derived from strain *F. graminearum* PH-1 [27, 28]. The *N*-terminal residue of compound **2** is *γ*-aminobutyric acid (GABA) unit, while it is replaced by guanidoacetic acid (GAA) in compound **3** [28, 29].

**Fig. 2:**
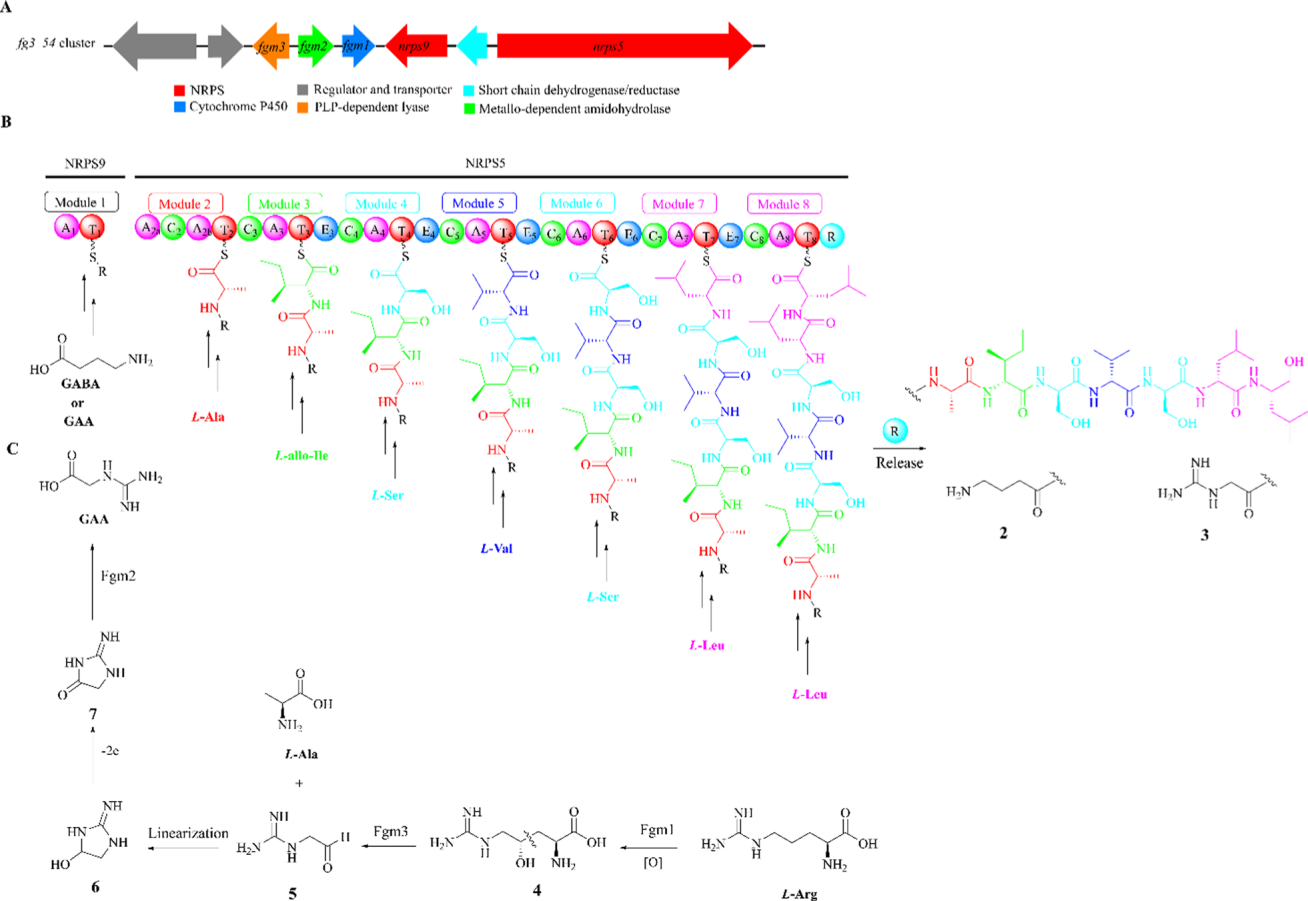
Biosynthetic pathway of fusaoctaxin A (**2**) and B (**3**). **A** The *fg3_54* cluster in *F. graminearum* PH-1; **B** Model of the assembly line for **2** and **3**. **C** Enzymatic biosynthesis for the formation of GAA

Two core NRPS genes *nrps5* and *nrps9* together with six adjacent genes located in the *fg3_54* cluster responsible for the biosynthesis of compounds **2** and **3** (Fig. 2A) were identified by laser microdissection and microarray approach [29, 30]. The essentiality of the *fg3_54* gene cluster was unambiguously verified through cluster deletion and individual knockout of several biosynthesis-associated genes including FG-Δ*nrps9*, and FG-Δ*nrps5*, FG-Δ*fgm4*, FG-Δ*fgm3* and FG-Δ*fgm1* [28]. The functions of the two key enzymes, NRPS9 and NRPS5, were further characterized by overexpression experiments [31]. The NRPS9 is a M1(A_1_-T_1_) di-domain protein that acts as a load module for initiating unit binding, while the NRPS5 harbors seven similar extension modules, M2(A_2a_-C_2_-A_2b_-T_2_)-M3(C_3_-A_3_-T_3_-E_3_)-M4(C_4_-A_4_-T_4_-E_4_)-M5(C_5_-A_5_-T_5_-E_5_)-M6(C_6_-A_6_-T_6_-E_6_)-M7(C_7_-A_7_-T_7_-E_7_)-M8(C_8_-A_8_-T_8_-R) and collaborates with the NRPS9 to biosynthesize octapeptides. These enzymes utilize GABA or GAA as a starting unit and extend the sequence with additional units including *L*-Ala, *L*-allo-Ile, *L*-Ser, *L*-Val and *L*-Leu residues (Fig. 2B) [32]. Each residue attached to the module containing the E domain (M3–M7) can undergo epimerization to acquire a *D*-configuration before transpeptidation. The peptidyl elongation was terminated by *L*-Leu through binding mediated by module M8, where the release (R) domain catalyzed a four-electron reduction to offload the octapeptide from the assembly line [29, 33].

Overexpression of genes *fgm1*, *fgm2* and *fgm3* along with their diverse combinations in *Pichia pastoris* GS115 showed these genes are responsible for the formation of GAA (Fig. 2C), which is a guanosine residue that serves as the initiating unit for the biosynthesis of compound **3**. *Fgm1*, *Fgm2* and *Fgm3* respectively encode cytochrome P450, metallo-dependent amidohydrolase, pyridoxal-5′-phosphate (PLP)-dependent lyase. Fgm1 oxidizes *L*-Arg to 4(R)-hydroxy-*L*-Arg (**4**), which selectively enables the activation of inert C4 atom by hydroxylation for subsequent C3-C4 cleavage [34]. Fgm3 catalyzes the cleavage of the C_*β*_-C_*γ*_ bond in **4** to produce **5** and *L*-Ala [35]. Fgm2 effectively hydrolyzes glycociamidine (**6**) to produce linearized GAA. The pathway for GAA formation in *F. graminearum* differs significantly from the well-known pathway that utilizes the *L*-Arg:*L*-Gly aminidotransferase (AGAT) to transfer amino group between *L*-Arg and *L*-Gly residues. Instead, it relies on *L*-Arg as a precursor through a series of chemical reactions including inert C−H bond activation, selective C−C bond cleavage, cyclization-based alcohol dehydrogenation, and amidohydrolysis-associated linearization [36].

### Gramillin

Gramillins A (**8**) and B (**9**) are two host-specific virulence factors initially isolated from several *F. graminearum* strains [37]. They possess a fused bicyclic structure in which the main peptide ring is cyclized through the carboxylic group of glutamic acid and the side chain of 2-amino adipic acid [38–40]. It was the first occurrence of anhydride bond being involved in the cyclization of a cyclic peptide [37, 41].

**Fig. 3:**
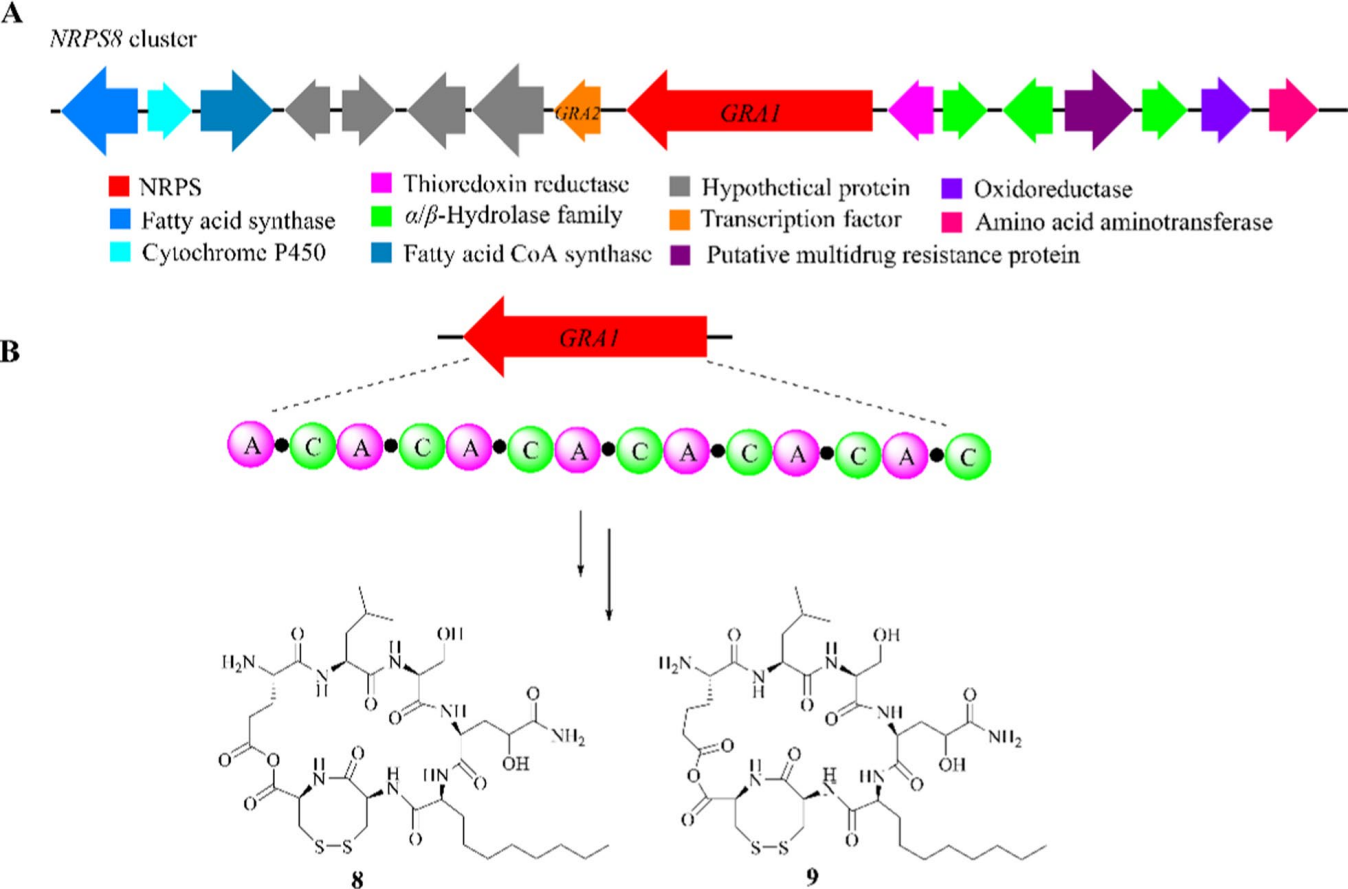
The biosynthetic logic for gramillins A (**8**) and B (**9**). **A** The *NRPS8* gene cluster in *F. graminearum*; **B** proposed biosynthesis of compounds **8** and **9**

The functions of the *NRPS8* gene cluster were determined through targeted gene disruption [42]. Gene *GRA1* encodes a multi-modular NRPS synthase that contains seven A and C domains [43]. *GRA2* encodes a transcription factor (TF) and is responsible for the regulation of cyclic peptide production (Fig. 3A) [44, 45]. By combining the Stachelhaus model and analyzing the conservation of the two adjacent A domains, the probable pathway for gramillins biosynthesis was identified. The biosynthetic pathway begins with Glu or 2-amino adipic acid and sequentially connects to Leu, Ser, *HO*-glutamine (*HO*-Gln), 2-amino decanoic acid, cysteine B (Cys B), and Cys A via other modules (Fig. 3B) [46, 47]. However, the functions of the other genes still need to be confirmed through additional specific experiments.

### Chrysogine

Chrysogine (**10**) is a natural pigment that was first obtained and studied in *Penicillium chrysogenum* [48]. Although this substance does not possess remarkablely biological property, its core scaffold, 4(3H)-quinazolinone, is the primary functional group in various first-line antitumor or sedative agents such as idelalisib, raltitrexed, and methaqualone and other marketed drugs (e.g. nolatrexed, albaconazole, and halofuginone) for treatment of malarial, inflammatory, HIV and diabetic diseases [49–52].

**Fig. 4:**
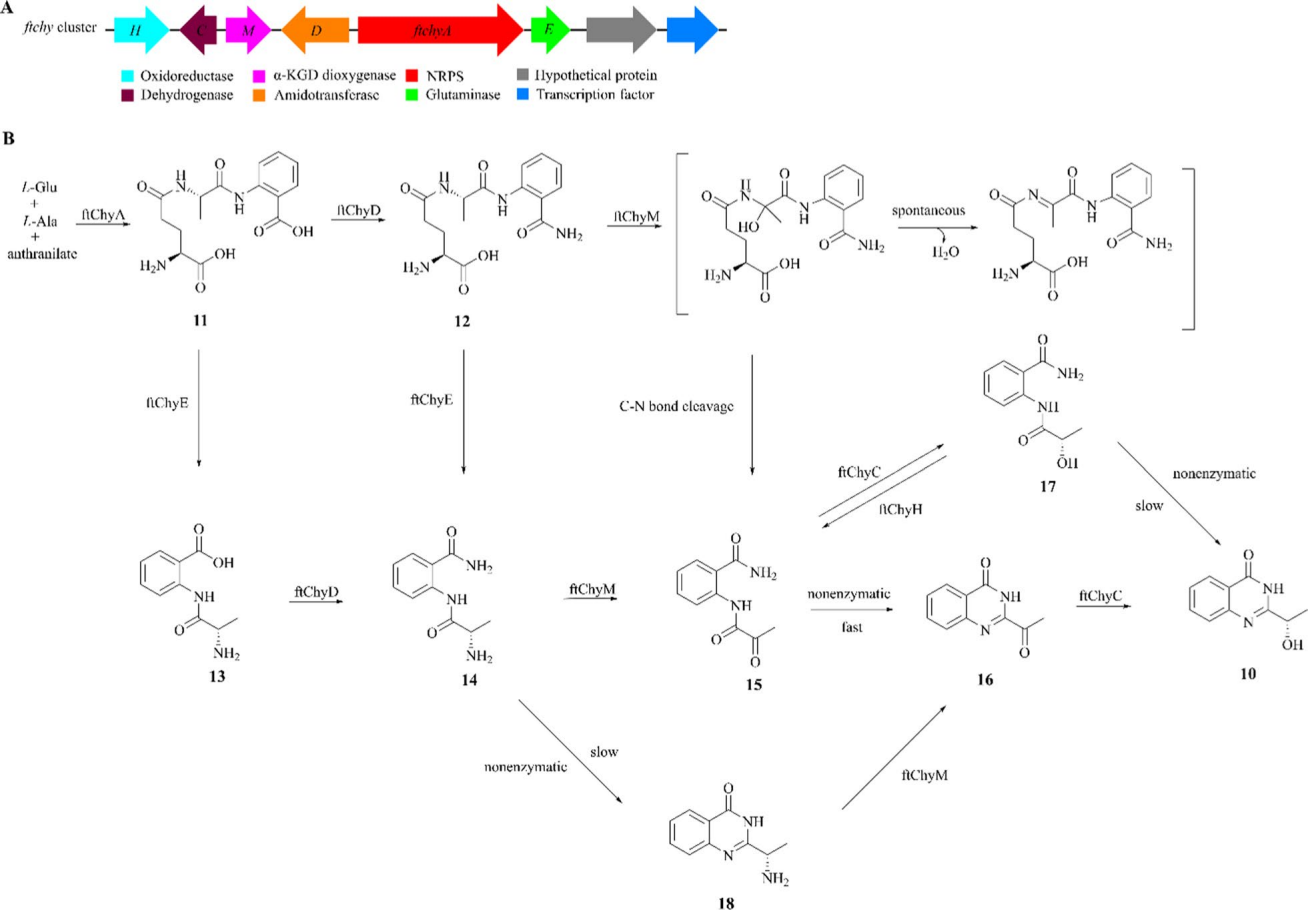
The proposed complex pathways for generating chrysogine (**10**). **A** The *ftchy* gene cluster in *F.tricinctum* CGMCC 3.4731; **B** the biosynthetic pathway for **10**

In the past decade great progress had been made in the biosynthetic investigation of **10** in *F. tricinctum* CGMCC 3.4731, which offers an alternative synthetic pathway for constructing the 4(3H)-quinazolinone scaffold [50, 53]. A highly homologous NRPS gene cluster named *ftchy* (Fig. 4A) was identified and confirmed to be responsible for the formation of **10** through heterologous expression in *Aspergillus nidulans* and in vitro incubation experiments in *E. coli* [50, 54, 55]. The results also indicated that gene *ftchyA* encodes a fungal two-module NRPS (ftChyA) for the biosynthesis of **11**, and the genes *ftchyC*, *ftchyD*, *ftchyE*, *ftchyH*, and *ftchyM* respectively encode a dehydrogenase (ftChyC), an amidotransferase (ftChyD), a tripeptide hydrolase (ftChyE), a flavin-dependent oxidase (ftChyH), and α-ketoglutaratedependent dioxygenase (α-KGD; ftChyM) [56, 57]. The enzyme ftChyD catalyses the amidation of **11** to **12** and **13** to **14** by utilizing inorganic ammonium ions or amides of *L*-Gln and ftChyE transforms **12** to **14** [48]. An unfamiliar α-KGD (ftChyM) catalyses the oxidative cleavage of the C-N bond for the production of **15** from **12**. The oxidase ftChyH only catalyses the dehydrogenation reaction and corrects the additional reduction of ftChyC towards **15**, ensuring the primary pathway **(15 → 16**) in the rapid construction of the 4(3H)-quinazolinone scaffold. These additional branching pathways depended on the nonenzymatic cyclization of ftChyM (**17 → 10**) or promiscuous substrate selectivity (**18 → 16 → 10**) (Fig. 4B).

### Beauvericin

Beauvericin (BEA, **19**) is a cyclic hexadepsipeptide that consists of a repetitive linkage between a *D*-hydroxy-isovaleryl (*D*-Hiv) and an N-methyl-phenylalanyl residue. It was firstly obtained from *Beauveria bassiana* and commonly discovered in several pathogenic *Fusarium* spp. [58, 59]. Bioassay results suggested that this alkaloid displays a wide range of biological activities including cytotoxic, apoptotic, anti-inflammatory, antimicrobial, and nematicidal activities [60–66].

A deeper understanding of the compound **19** biosynthesis gene cluster (*bea* cluster) in *F. proliferatum* LF061 was achieved by knocking out the specified genes using *Agrobacterium* AGL-1 mediated transformation (ATMT) protocol [67, 68]. A gene of 9413 bp (*BEA1*) responsible for encoding a hexadepsipeptide synthetases (NRPS22) was revealed, and the *kivr* gene encodes a novel NADPH-dependent 2-ketoisovalerate reductase (KIVR) responsible for the metabolism of pyruvate to *D*-Hiv was also unveiled [69]. Sequence analysis of other genes showed that *orf1*, *orf3*, *orf4*, *orf5*, *orf6*, and *orf10* respectively encode putative thioesterase, triacylglycerol lipase, chitinase, zinc-dependent metalloproteinase, furinase, and multidrug transporter [70, 71].

**Fig. 5:**
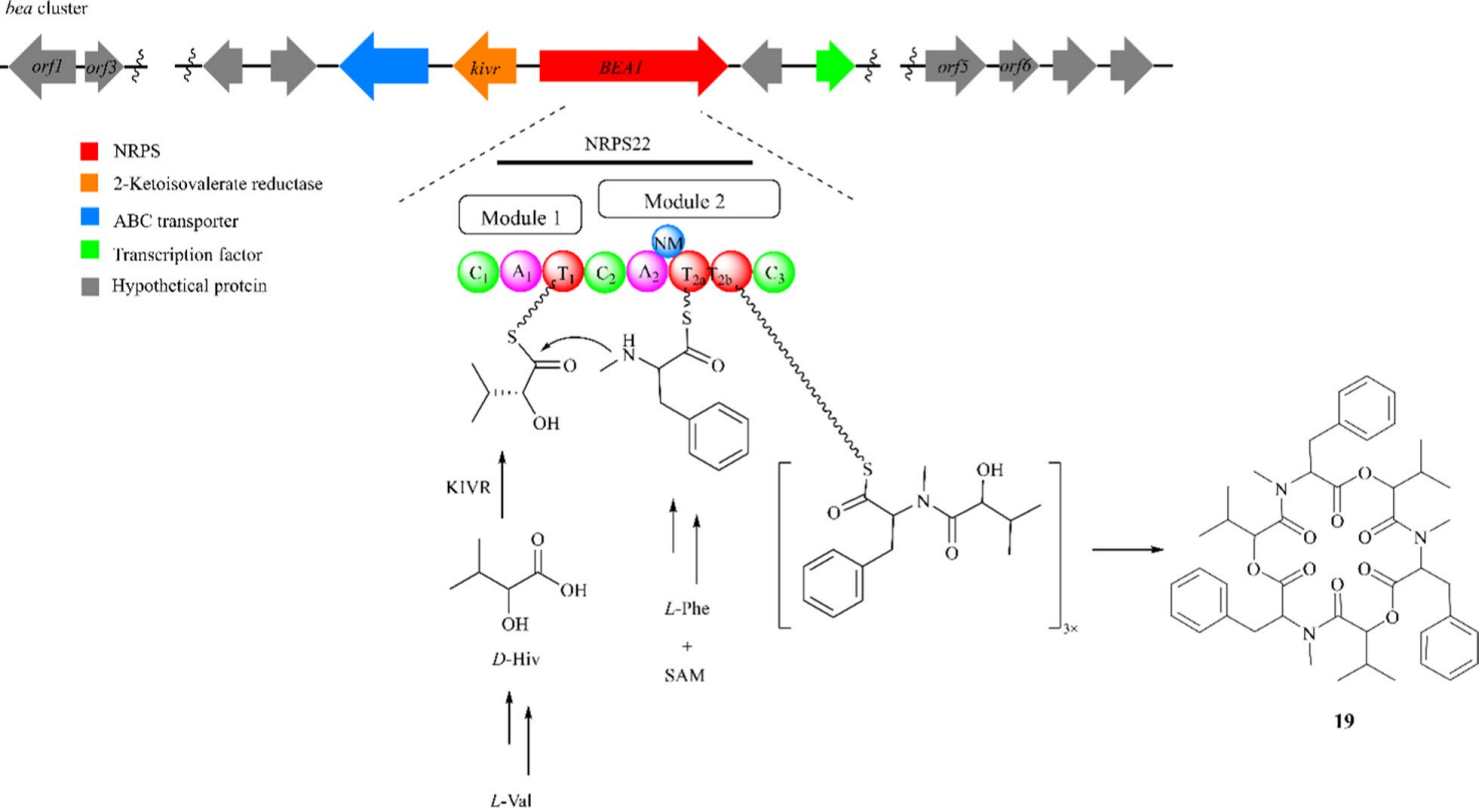
The scheme of BEA (**19)** biosynthesis and the *bea* cluster in *F. proliferatum* LF061

The small two-gene cluster for BEA biosynthesis in strain LF061 consists of an NRPS gene and a KIVR-encoding gene [72]. *D*-Hiv is recognized by the A_1_ domain in module 1 of NRPS22 and attached to the T_1_ domain as a thioester. *L*-Phe is specifically activated by the A_2_ domain and is loaded to the twin T_2_ domain in module 2. An integrated N-methyltransferase domain is also present in NRPS22, which is responsible for the methylation of the *L*-Phe residue (Fig. 5) [67, 71]. This serves as a classic example of acting through the core NRPS synthase and provides valuable insights for subsequent studies [60].

### Sansalvamide A

Sansalvamide A (**20**) is a cyclic pentadepsipeptide composed of an α-hydroxyisocaproic acid (α-HICA) unit and four protein amino acids (*L*-Val, *L*-Leu, *L*-Phe, *L*-Leu). It was originally discovered in the crude extract of an unknown *Fusarium* strain, which was collected from the surface of the seagrass *Halodule wrightii* [73–75]. Bioassay tests indicated that compound **20** is an effective cytotoxin in the colon cancer cell lines COLO 205 and HCT116 and the melanoma cell line SK-MEL-2 [75, 76].

**Fig. 6:**
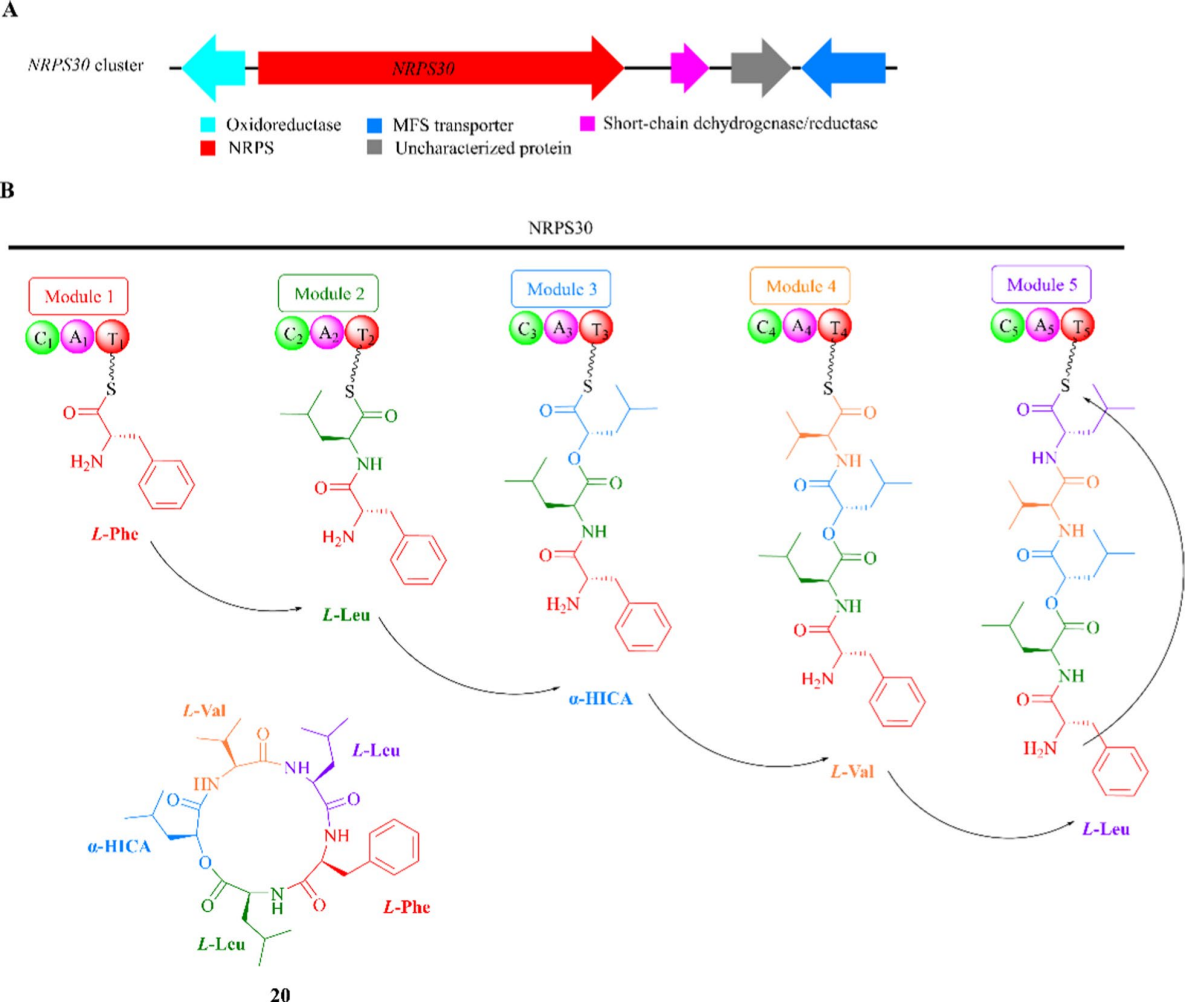
The proposed biosynthetic pathway for sansalvamide A (**20**). **A** The *NRPS30* cluster in *Fusarium solani* FGSC 9596; **B** the compound **20** biosynthesis logic

The BGC *NRPS30*, which is responsible for the formation of compound **20** in *F. solani* FGSC 9596, was characterized through a gene knockout experiment using the ATMT approach [77, 78]. This cluster contains at least four genes that encode NRPS30 synthetase (gene *NRPS30*), oxidoreductase, short-chain dehydrogenase/reductase, and MFS transporter (Fig. 6A). Among the five modules of the NRPS30 enzyme, only the first amino acid of the A_3_ domain is glycine, while the remaining four are aspartic acid [46, 79]. This suggests that α-HICA is loaded as the third substituent during the biosynthesis of compound **20**, as the lack of an acidic residue in the first position is only observed for A domain with non-amino acid substrates [80]. NRPS30 utilizes *L*-Phe as a starting unit and extends the sequence with additional units, including *L*-Leu, α-HICA, *L*-Val, and *L*-Leu (Fig. 6B).

### Apicidin F

Apicidin F (APF, **21**) is a cyclic tetrapeptide produced by *F. fujikuroi* [81]. Structurally, APF consists of N-methoxy-*L*-tryptophan (**25**), *L*-2-aminooctanedioic acid (**26**), *D*-pipecolic acid (*D*-pip; **23**) and *L*-phenylalanine [82, 83]. Biological evaluation showed that this compound has the ability to inhibit histone deacetylase and is a therapeutic agent for antimalarial treatment against *Plasmodium falciparum* [84, 85].

**Fig. 7:**
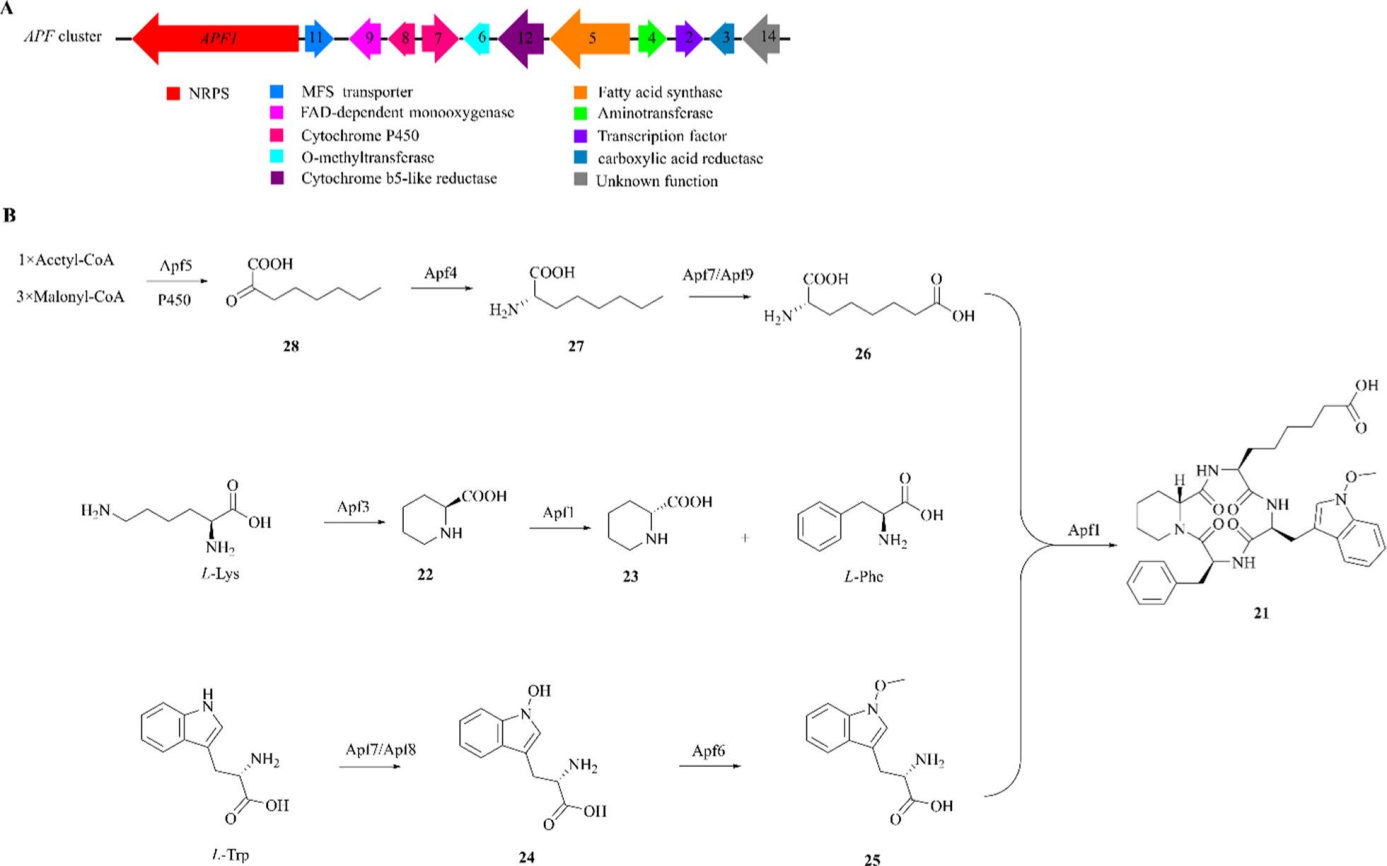
Proposed biosynthetic pathway of APF (**21**) **A** The *APF* gene cluster in *F. fujikuroi* IMI58289; **B** The biosynthesis logic of APF (**21**)

A highly homologous NRPS gene cluster named *APF* was uncovered through homologous comparison and genomic sequence analysis (Fig. 7A) [86, 87]. Further exploration of the *APF* cluster and targeted gene replacement of *APF1* revealed that Apf1, a key NRPS enzyme, is responsible for the biosynthesis of compound **21** [88–90]. The deletion of other functional genes suggested that the *APF* gene cluster consists with *APF2*, *APF3*, *APF4*, *APF5*, *APF6*, *APF7*, *AFP8*, *APF9*, *APF11*, and *APF12*, which respectively encode a transcription factor (Apf2), a putative Δ1-pyrroline-5-carboxylic acid reductase (Apf3), an aminotransferase (Apf4), a fatty acid synthase (Apf5), an O-methyltransferase (Apf6), two cytochrome P450 oxidases (Apf7/Apf8), a FAD-dependent monooxygenase (Apf9), a MFS transporter (Apf11), and a cytochrome b5-like reductase (Apf12).

The comparison of metabolite profile of the knockout mutants revealed that only six genes (*APF1*, *APF3*, *APF4*, *APF5*, *APF6*, *APF7*/*AFP8*/*APF9*) directly participate in the biosynthesis of APF [85]. Apf3 reduces* L*-lysine to *L*-piperidinic acid (**22**), which is subsequently converted to **23** by Apf1. *L*-tryptophan is initially oxidized to N-hydroxyl-*L*-tryptophan (**24**) by one of the two P450 enzymes (Apf7/Apf8), followed by conversion to **25** by Apf6. Apf5 is responsible for the condensation of three malonyl-CoA units and an acetyl-CoA into the octanoic acid backbone, which is then oxidized to form **28** by a P450 oxygenase. Apf4 catalyzes the exchange of the keto group of **28** with the amino group to form **27**. Apf7/Apf9 may be involved in the conversion of **27** to **26**. Ultimately, APF is generated by combining the four precursors in the presence of Apf1 (Fig. 7B). This represents a unique case of NRPS synthase function, where the NRPS enzyme is not fully functional until the final step.

### Fusarochromene (NRPS-like)

Fusarochromene (**29**) firstly isolated from *F. sacchari* has structural similarities to fusarochromanone (**30**), which is a lead compound for cancer treatment [91, 92]. Compound **30** demonstrates a wide range of biological activities, such as angiogenesis inhibition, prevention of cell reproduction, and induction of apoptosis in numerous cancer cells, especially COS7 and HEK293 cells [93, 94].

**Fig. 8:**
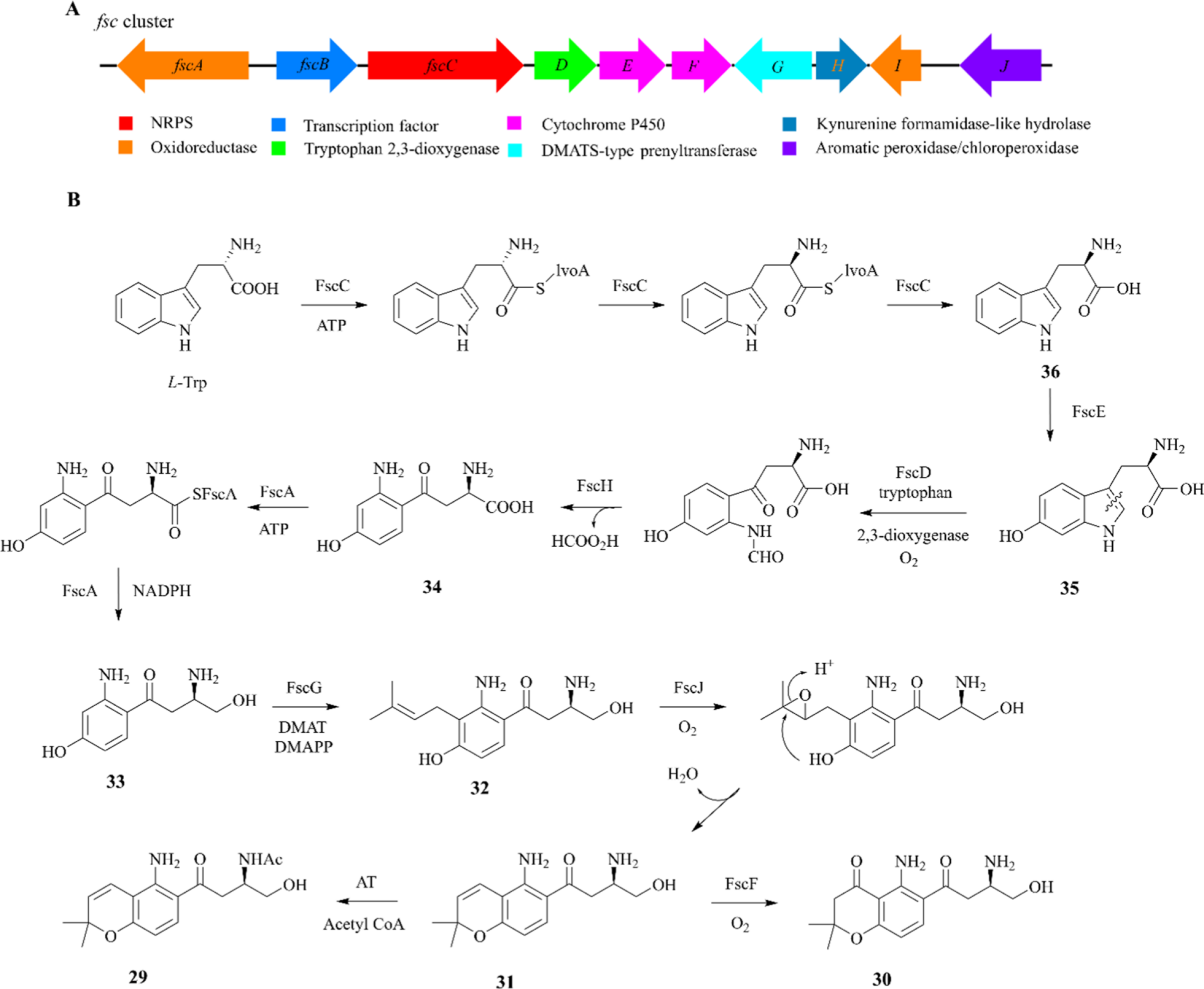
The putative biosynthetic pathway for fusarochromene (**29**) and fusarochromanone (**30**). **A** The *fsc* cluster identified in the genome of *F. equiseti*; **B** proposed assembly path to compounds **29** and **30**

Retro-biosynthetic analysis and ^13^C-labelled tryptophan experiments suggested that compounds **29** and **30** were actually obtained through oxidative cleavage of tryptophan [91]. The *fsc* gene cluster was identified by searching the genome of *F. equiseti* for potential tryptophan dioxygenase (TDO) and dimethylallyl diphosphate transferase (DMAT) genes. Through homologous comparison, the functions of these genes showed that *fscA*, *fscB*, *fscC*, *fscD*, *fscE*, *fscF*, *fscG*, *fscH*, *fscI*, and *fscJ* respectively encode two oxidoreductases (FscA, FscI), a TF (FscB), an NRPS-like enzyme (FscC), a dioxygenase (FscD), two P450 enzymes (FscE, FscF), a DMAT enzyme (FscG), a kynurenine formamidase-like hydrolase (FscH), and an aromatic peroxidase/chloroperoxidase (FscJ) (Fig. 8A) [95, 96].

A biosynthetic pathway for **29** and **30** is proposed in Fig. 8B. *L*-tryptophan is converted to *D*-tryptophan (**36**) in the presence of FscC, and subsequently hydroxylated by FscE to yield 6-hydroxytryptophan (**35**) [97]. The pyrrole ring undergoes cleavaged by FscD and is finally converted to 4-hydroxykyrunenine (**34**). FscA reduces the carboxyl group to primary alcohol (**33**) and FscG, a DMATS-type prenyltransferase, performs prenylation to **32** with the formation of a chromene ring. **32** is catalyzed by FscJ, leading to the formation of desacetyl-fusarochromene (**31**). Epoxidation (FscF) and rearrangement reactions of chromene double bonds convert compound **31** to **30**. Although specific acetyltransferases were not found near the *fsc* BGC, several predicted enzymes containing the N-acetyltransferase superfamily domain were discovered in the genome of *F. equiseti*. These predicted enzymes may have the potential to convert compound **31** to **29** [98].

## Hybrid PKS-NRPS products

Polyketide synthase (PKS) and NRPS hybrid systems typically rely on intricate protein–protein interactions to enable the seamless transfer of intermediates between these multimodular enzymes [99–102]. The PKS in *Fusarium* strain includes the *β*-keto synthase (KS) domain, the acyltransferase (AT) domain, the *β*-keto reductase (KR) domain, dehydrogenase (DH) domain, methyltransferase (MT) domain, enoyl reductase (ER) domain and acyl carrier protein (ACP) domain.

### Fusaristatin A

Fusaristatin A (**37**) is a lipopeptide composed of three amino acid residues (glutamine, dehydroalanine, and *β*-aminoisobutyric acid) along with their attached polyketide chains. It was originally separated from *Fusarium* sp*.* YG-45 and lately detected in *Phomopsis longicolla* S1B4 and other *Fusarium* strains including *F. graminearum*, *F. avenaceuma* and *Fusarium* sp*.* FN080326 [103–107]. Cytotoxic assay indicated that compound **37** displays growth-inhibitory activity against lung cancer cells LU 65 with an IC_50_ value of 23 μM [103, 108].

**Fig. 9:**
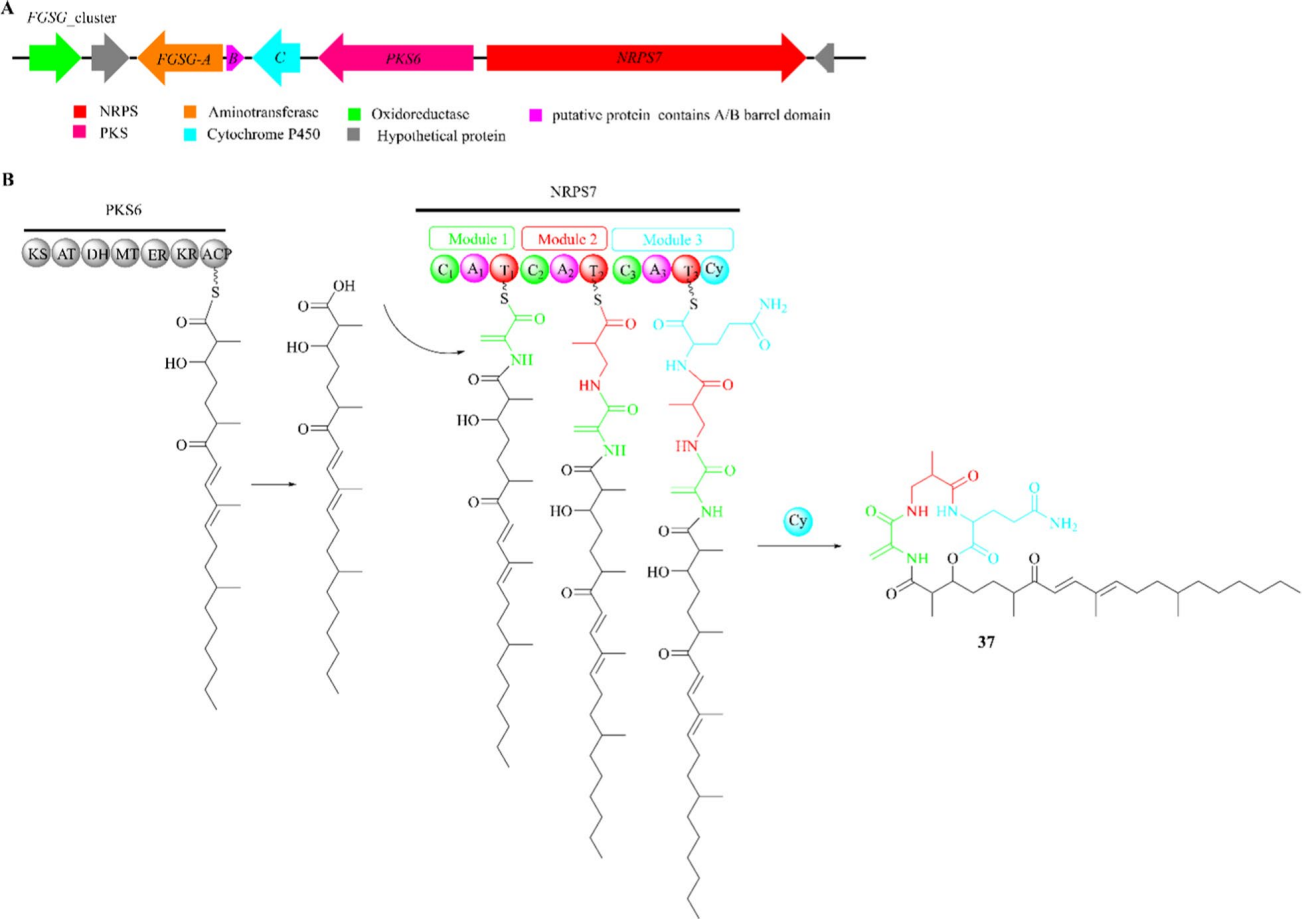
Proposed biosynthetic pathway of fusaristatin A (**37**). **A** The *FGSG* gene cluster in *F. graminearum*; **B** The PKS6 and NRPS7 collaborative model of the biosynthetic logic of **37**

As shown in Fig. 9A, the *FGSG* cluster in *F. graminearum* consists of at least five genes: *PKS6*, *NRPS7*, *FGSG-A*, *FGSG-B*, and *FGSG-C*. Deletion of *NRPS7/PKS6* resulted in the absence of **37**, confirming that PKS6 and NRPS7 are the two key enzymes jointly responsible for its production. Additionally, *FGSG-C* is predicted to encode a cytochrome P450 monooxygenase, *FGSG-A* encodes an aminotransferase, and *FGSG-B* encodes a putative protein containing a stress response A/B barrel domain [108]. The biosynthetic pathway of product **37** is mainly accomplished by PKS6 and NRPS7. As the *FGSG* cluster lacks acyltransferases, the polyketide synthesized by PKS6 is directly transferred to NRPS7. Then module 1–3 of NRPS7 sequentially adds Ala, Gln, and *β*-aminoisobutyric acid, and is finally released through cyclization (Fig. 9B). Although the* β*-aminoisobutyric acid units are most likely not freely available to the NRPS7, the *FGSG* cluster harbors cytochrome P450 and aminotransferases, which could potentially obtain it from thymidine.

### W493 B

W493 B (**38**) is a lipopeptide consisting of six amino acid residues [*D*-allo-Thr, *L*-Ala, *D*-Ala, *L*-Gln, *D*-Tyr, and *L*-valine/isoleucine (Val/Ile)], which are linked to a polyketide chain of 3-hydroxy-4-methyltetradecanoic acid. It was initially isolated from *Fusarium* sp. and displayed inhibitory effect on the growth of *Venturia inaequalis*, *Monilinia mali*, and *Cochliobolus miyabeanus* [109, 110].

**Fig. 10:**
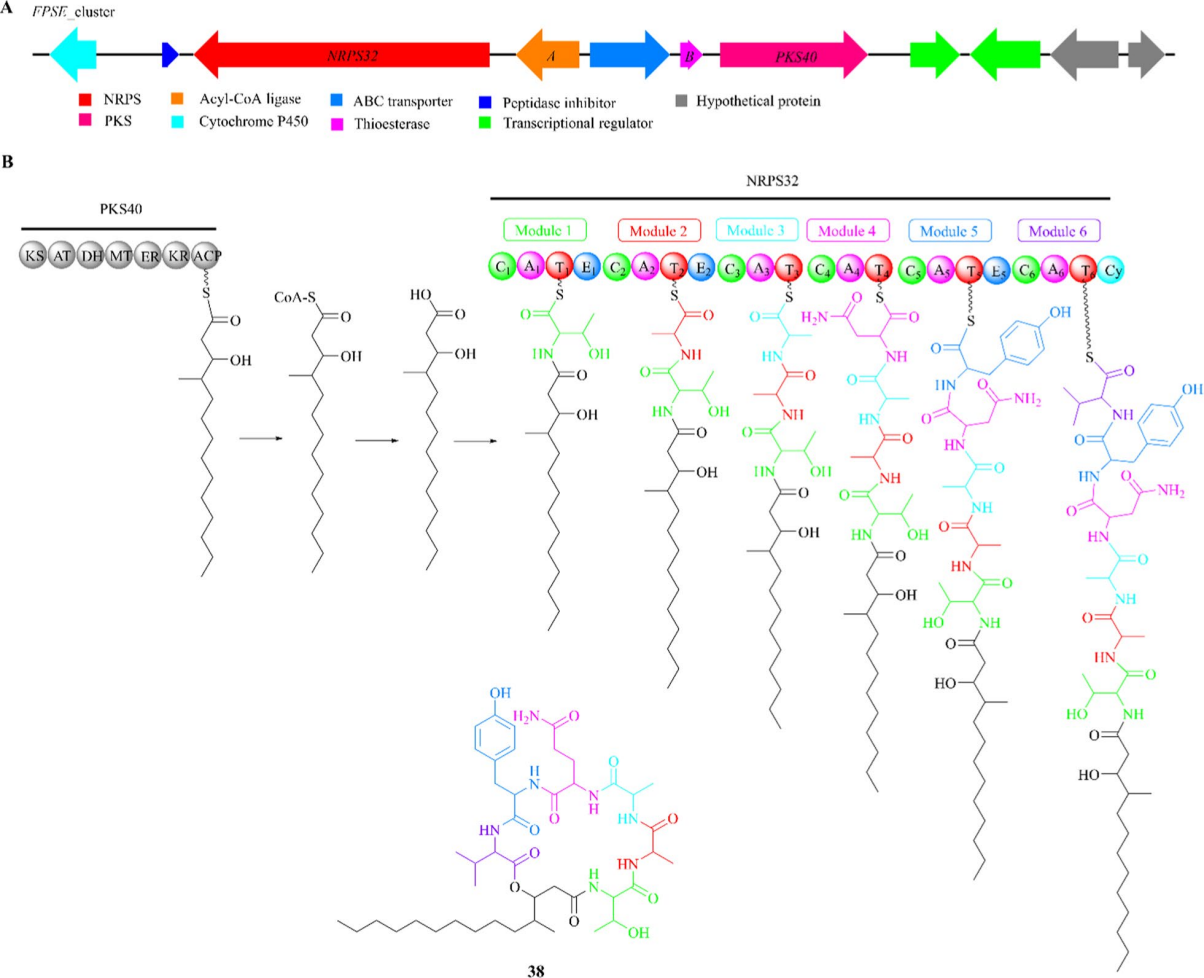
The proposed biosynthetic pathway of W493 B (**38**). **A** The *FPSE* gene cluster in *F. pseudograminearum*; **B** the PKS40 and NRPS32 collaborative model of the biosynthetic logic of **38**

The *FPSE* cluster, consisting of at least four genes (*PKS40*, *NRPS32*, *FPSE-A*, *FPSE-B*), was identified in *F. pseudograminearum* through the analysis of the conserved genes [108]. These genes were respectively predicted to encode a PKS enzyme (PKS40), a NRPS enzyme (NRPS32), an acyl-CoA ligase and a thioesterase (Fig. 10A). The biosynthetic pathway of W493 B is primarily catalyzed by PKS40 and NRPS32, which respectively play important roles in the formation of 4-methyltridecanoic acid thioester and a hexapeptide (Fig. 10B). The T_1_ domain of NRPS32 is responsible for accepting threonine, which is adenylated by the A_1_ domain and then combined with *D*-allo-threonine formed by the E_1_ domain. Five consecutive modules bind Ala, Ala, Gln, Tyr, and Val/Ile to form the final product and release it through the cyclization domain [108]. The biosynthetic pathways of compounds **37** and **38** provide a comprehensive overview of lipopeptide biosynthesis.

### Fusaric acid

Fusaric acid (FA, **39**), formed by adding a butyl group to the 5-position C of 2-picolinic acid, is a mycotoxin produced by numerous *Fusarium* species, including *F. oxysporum*, *F. heterosporum*, *F. verticillioides*, and *F. fujikuroi* [111, 112]. FA is a broad-spectrum plant toxin with high phytotoxicity, and exhibits potent acanthamoebicidal activity and inhibits HIV-1 tat-induced transactivation and apoptosis [113–117].

**Fig. 11:**
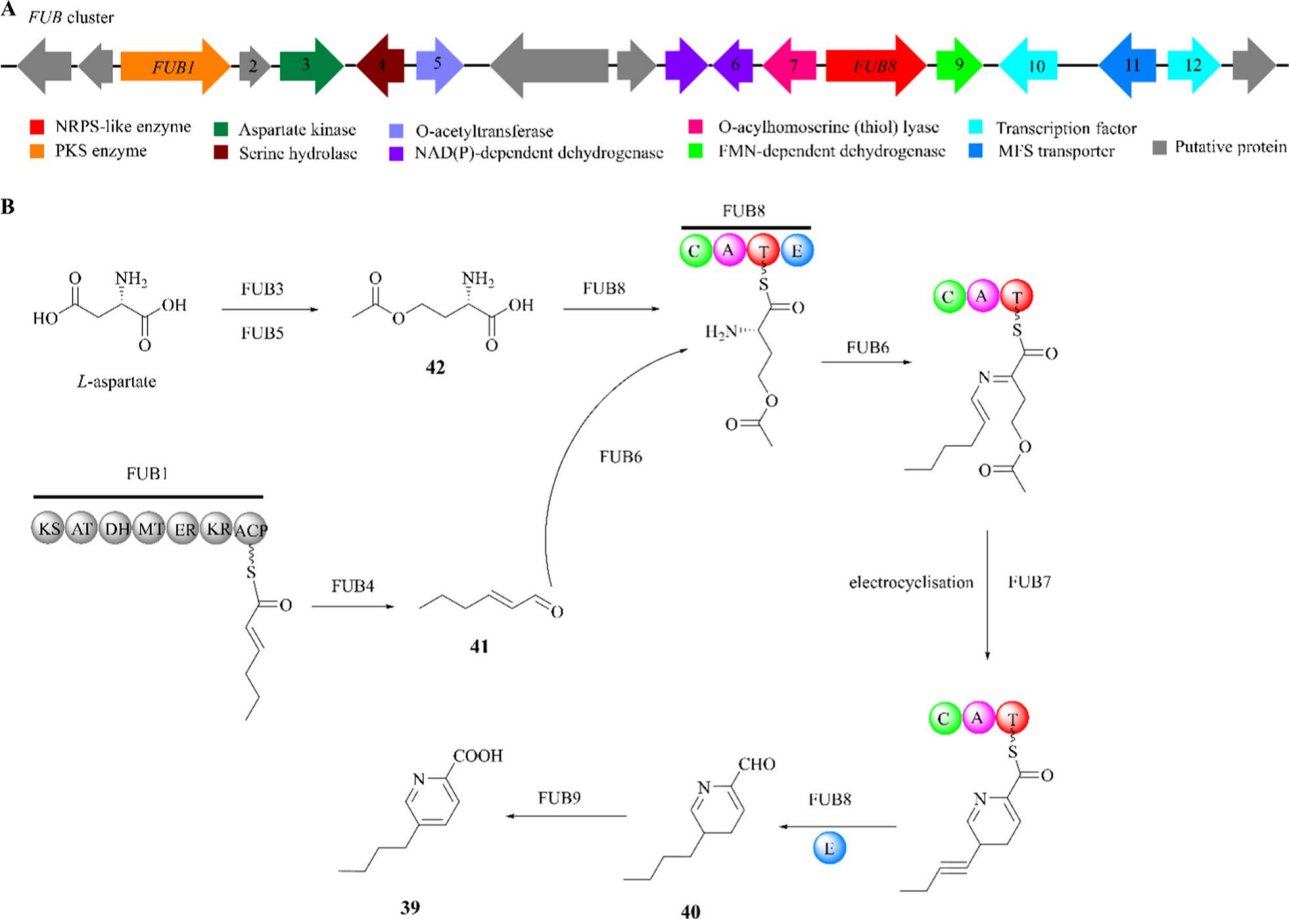
The proposed biosynthetic pathway of fusaric acid (**39**). **A** The *FUB* gene cluster in *F. fujikuroi* IMI58289; **B** the fusaric acid biosynthesis logic

The *FUB* cluster in *F. fujikuroi* was identified through targeted gene deletion, complementation, and overexpression experiments (Fig. 11A) [118–120]. These experiments suggest that a total of 12 genes are responsible for FA biosynthesis [121]. As illustrated in Fig. 11A, the functions of these genes showed that *FUB1-12* respectively encode a PKS enzyme (FUB1), a putative protein (FUB2), an aspartate kinase (FUB3), a serine hydrolase (FUB4), a homoserine O-acetyltransferase (FUB5), a NAD(P)-dependent dehydrogenase (FUB6), an O-acylhomoserine (thiol) lyase (FUB7), an NRPS-like enzyme (FUB8), a flavin mononucleotide (FMN)-dependent dehydrogenase (FUB9), two fungal-type Zn(II)2Cys6 transcription factors (FUB10 and FUB12), and a MFS transporter (FUB11) [122, 123].

The FA biosynthetic pathway has been proposed in Fig. 11B. With the combined action of FUB3 and FUB5, *L*-aspartate is converted to O-acetyl-homoserine (**42**). FUB1 generates the triketide trans-2-hexenal (**41**), which is potentially released by FUB4 and linked to the NRPS-bound amino acid precursor by Fub6. After further modification by FUB7, the NRPS-bound amino acid precursor is released by FUB8 to form **40**, which is finally oxidized by FUB9 to form FA.

## Hybrid PKS/NRPS products

The compounds generated by PKS/NRPS hybrid megaenzymes are especially intriguing due to their structural complexity [124, 125]. This hybrid megaenzymes consists of an NRPS module and a PKS module together.The PKS module synthesizes the linear polyketide backbone, which is released after ligating with amino acids through the action of the NRPS module [126–129]. It is then further converted to more complex metabolites by oxidase or other enzymes.

### Fusarin C

Fusarin C (**43**), a representative of substituted 2-pyrrolidinone metabolites, was firstly isolated in *F. moniliforme* and is widely present in *Fusarium* spp., including *F. graminearum*, *F. oxysporum*, *F. verticillioides* and *F. fujikuroi* [130–135]. Biological assays suggested that compound **43** acts as an estrogenic agonist, which stimulates the growth of the breast cancer cell line MCF-7 in concentrations ranging from 0.1 to 20 μM and inhibits its growth in concentrations exceeding 50 μM [136, 137]. Interestingly, **43** was found to induce esophageal and forestomach carcinoma in mouse and rat models, while this effect was not observed by Gelderblom and co-workers [138–141].

**Fig. 12:**
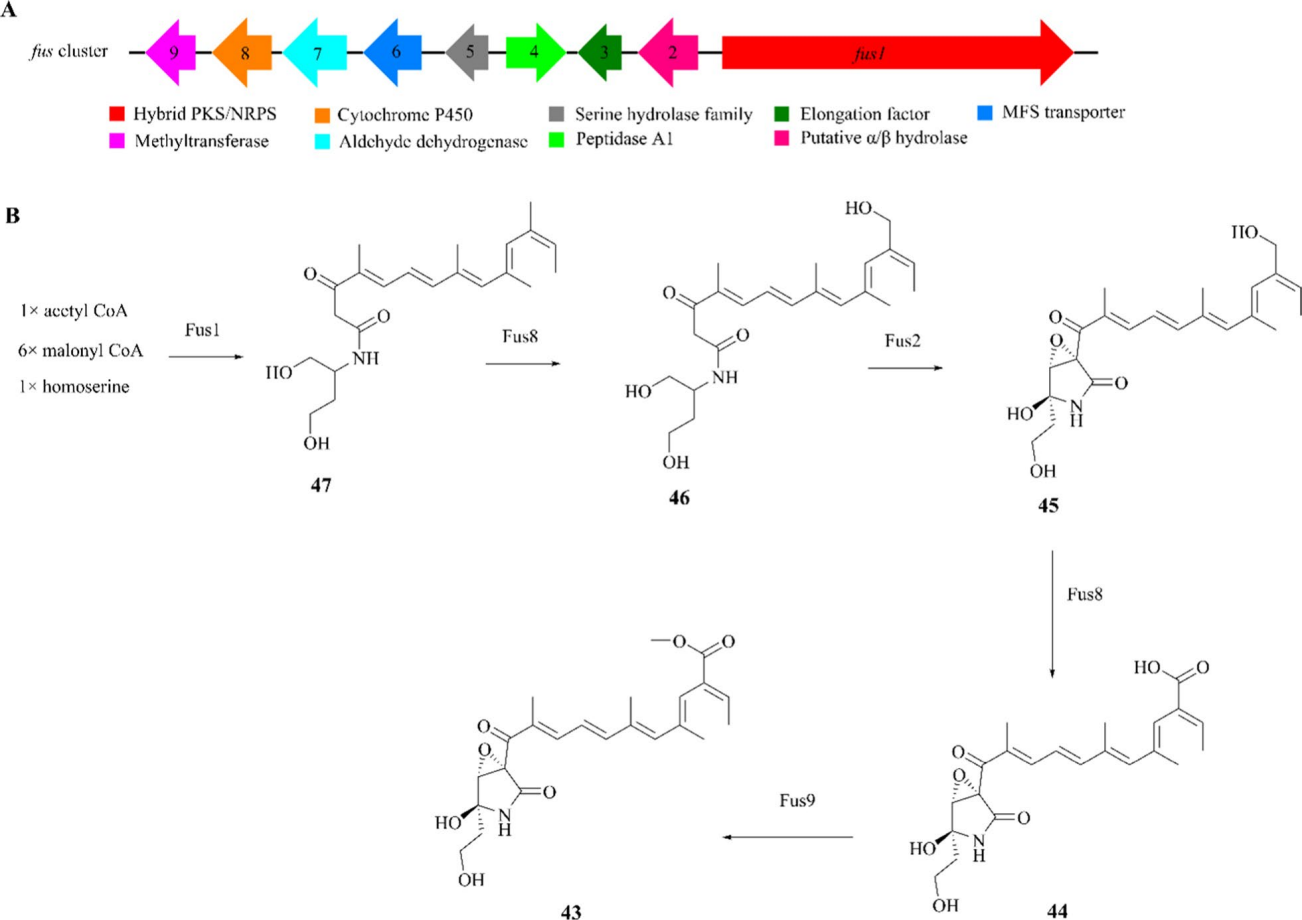
Proposed biosynthetic pathway of fusarin C (**43**). **A** The *fus* gene cluster in *F. fujikuroi* IMI58289; **B** the biosynthesis logic of **43**

Gene knockout experiment showed that the *fus* cluster in *F. fujikuroi* consists of nine coregulated genes, of which *fus2*-*fus9* are adjacent to gene *fus1* (the hybrid PKS/NRPS; Fus1) [142–144]. Fus2 is related to a putative *α*/*β* hydrolase, which is probably involved in the 2-pyrrolidone ring formation. Deduced proteins show similarity to a subunit of elongation factor (Fus3), a peptidase A1 (Fus4), a serine hydrolase family (FSH; Fus5), a major facilitator superfamily transporter (MFS; Fus6), an aldehyde dehydrogenase (Fus7), a cytochrome P450 (Fus8), a characterized methyltransferase (Fus9) (Fig. 12A) [135].

The intermediates of compound **43** were only identified in the Δ*fus2*, Δ*fus8*, Δ*fus9*, and Δ*fus2-9* mutants, suggesting that the genes *fus3*, *fus4*, *fus5*, *fus6*, and *fus7* are largely uninvolved in the production of fusarin C. The proposed fusarin C biosynthetic pathway is as follows: Fus1 is responsible for the condensation of one acetyl-CoA with six malonyl-CoA and homoserine to form prefusarin (**47**). Fus8 then oxidizes **47** to form **46**, which is an essential reaction until Fus2 catalyzes the formation of 20-hydroxy-prefusarin (**45**). **45** is further oxidized to produce **44** by Fus8. The final step involves the methylation of the hydroxyl group of C-21 by Fus9, resulting in the production of fusarin C (Fig. 12B). The co-cultivation of different mutants and intermediates analysis further confirms that Fus1, Fus2, Fus8, and Fus9 are sufficient for the biosynthesis (see Additional file 1).

### Oxysporidinone

**Fig. 13:**
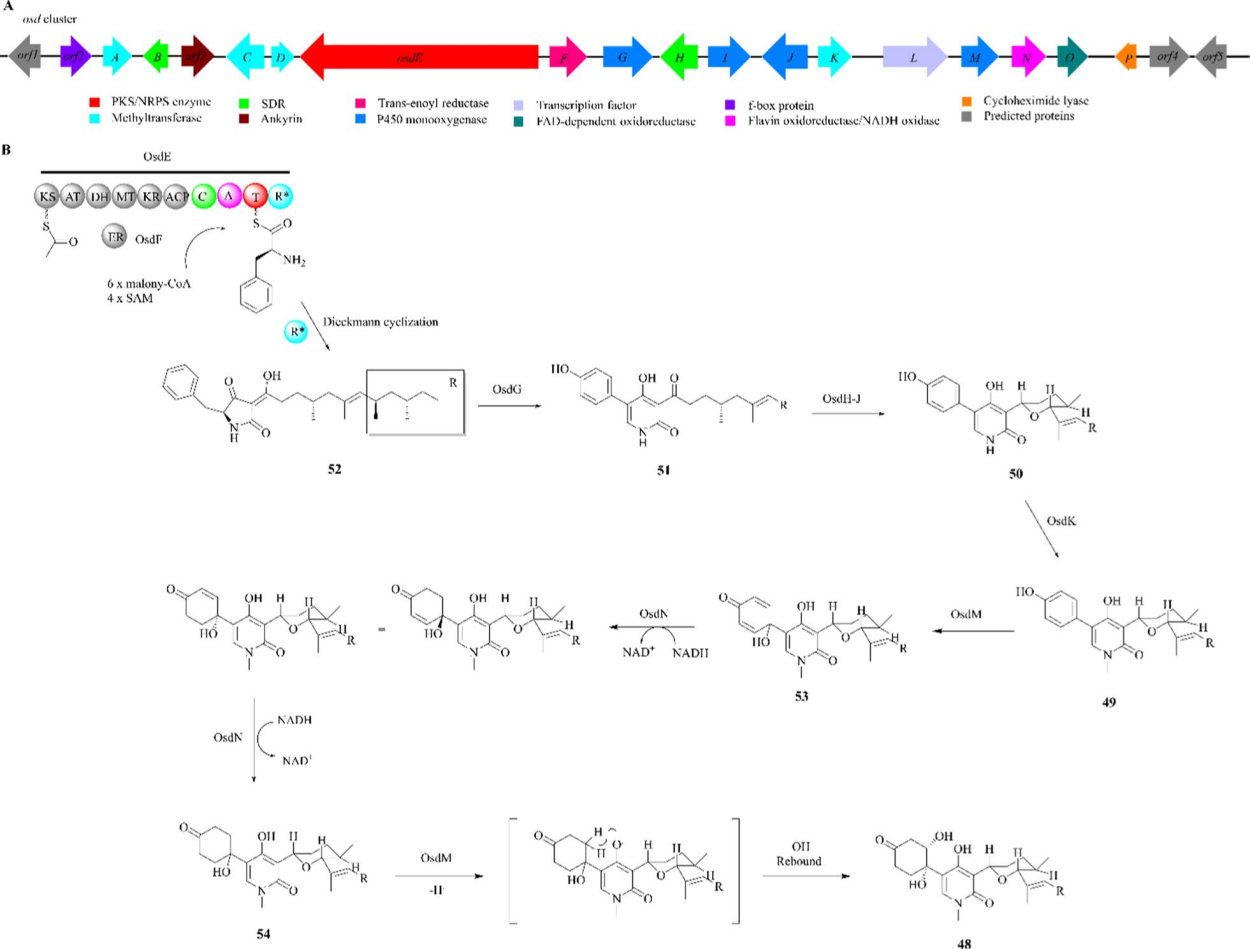
Proposed biosynthetic pathway of oxysporidinone (**48**). **A** The *osd* gene cluster in *F. oxysporum* ACCC 36465; **B** the scheme of the assembly line for **48**

Oxysporidinone (**48**), a novel antifungal product with 4-hydroxy-2-pyridone backbone and a unique hydroxy-substituted cyclohexane ring, was firstly isolated from *F. oxysporum* [145, 146]. The oxysporidinone biosynthesis gene cluster (*osd* cluster) was identified in *F. oxysporum* ACCC 36465 by regulator activation and gene knockout studies (Fig. 13A) [147]. The *osd* cluster, containing 21 putative encoding genes (*osdA*-*P* and *orf1-5*), includes a core PKS/NRPS hybrid enzyme (OsdE), a trans-enoyl reductase (OsdF), two short-chain dehydrogenases/reductases (SDR; OsdB and H), four methyltransferases (MT; OsdA, C, D and K), four P450 monooxygenases (OsdG, I, J and M), a fungus-specific transcription factor (OsdL), a flavin oxidoreductase/nicotinamide adenine dinucleotide (NADH) oxidase (OsdN), a flavin adenine dinucleotide (FAD)-conjugated oxidoreductase (OsdO), a cycloheximide lyase (OsdP), an ankyrin (ORF3), a f-box protein (ORF2) and three unknown proteins (ORF1,ORF4,ORF5).

The biosynthetic pathway of **48** was proposed through heterologous expression and in vitro enzyme assays [147–149]. In the presence of PKS/NRPS enzyme (OsdE) with OsdF, six malonyls and four SAMs combine to form the backbone structure of tetrameric acid (**52**). Compound **52** undergoes a classic ring-expansion reaction catalyzed by OsdG to produce 2-pyridone (**51)**. The formation from **51** to **50** is catalyzed by OsdH-J. OsdK is responsible for the N-methylation process, which converts **50** to form **49**. Compound **49** is then converted to **53** by OsdM, a TenA-like cytochrome P450 enzyme that oxidizes the phenol ring and forms a [6–5–6] ring system. OsdN carries out two consecutive reduction steps to produce **54**. Finally, OsdM adds another hydroxyl group to **54**, resulting in the formation of compound **48** (Fig. 13B). Two enzymes (OsdM, OsdN) repeatedly act on the phenol moiety in the substrate. This pathway enhances the current understanding of the mechanism of enzymatic phenol dearomatization.

### Fusaridione A

**Fig. 14:**
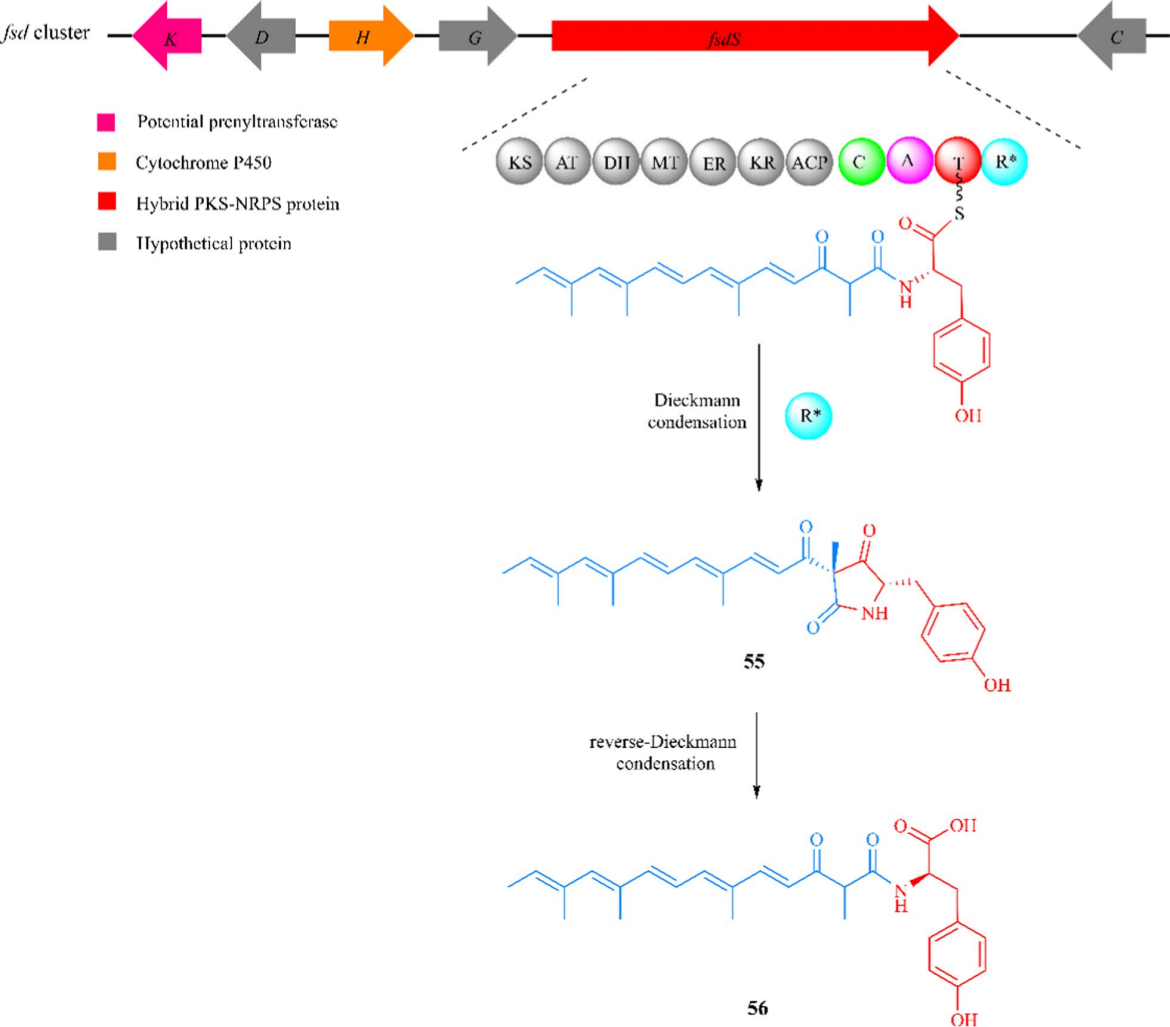
The *fsd* gene cluster in *F. heterosporum* ATCC 74349 and proposed biosynthetic logic of fusaridione A (**55**)

Fusaridione A (**55**) is an unstable tyrosine-derived 2,4-pyrrolidinedione produced by *F. heterosporum* [150–153]. Genomic analysis has revealed a silence gene, *fsdS*, which consists of a hybrid PKS and NRPS module. The putative biosynthesis pathway of fusaridione A was unveiled by *fsdS* gene knockout experiments [154]. The polyketide chain is first synthesized by the addition of seven acetyl-CoA units. Each extension requires the involvement of the KS, AT, KR, DH and ACP domain. Then, the tyrosine is activated and attached to the polyketide chain in the presence of the C, A and T domains. Compound **55** is finally released through the Dieckmann cyclase R* domain [16, 155]. The unstable pyrrolidinedione ring is opened by a reverse Dieckmann reaction, resulting in the formation of product **56** (Fig. 14) [156]. Further exploration is required to elucidate the genes that are closely related to gene *fsdS*.

### Equisetin

**Fig. 15:**
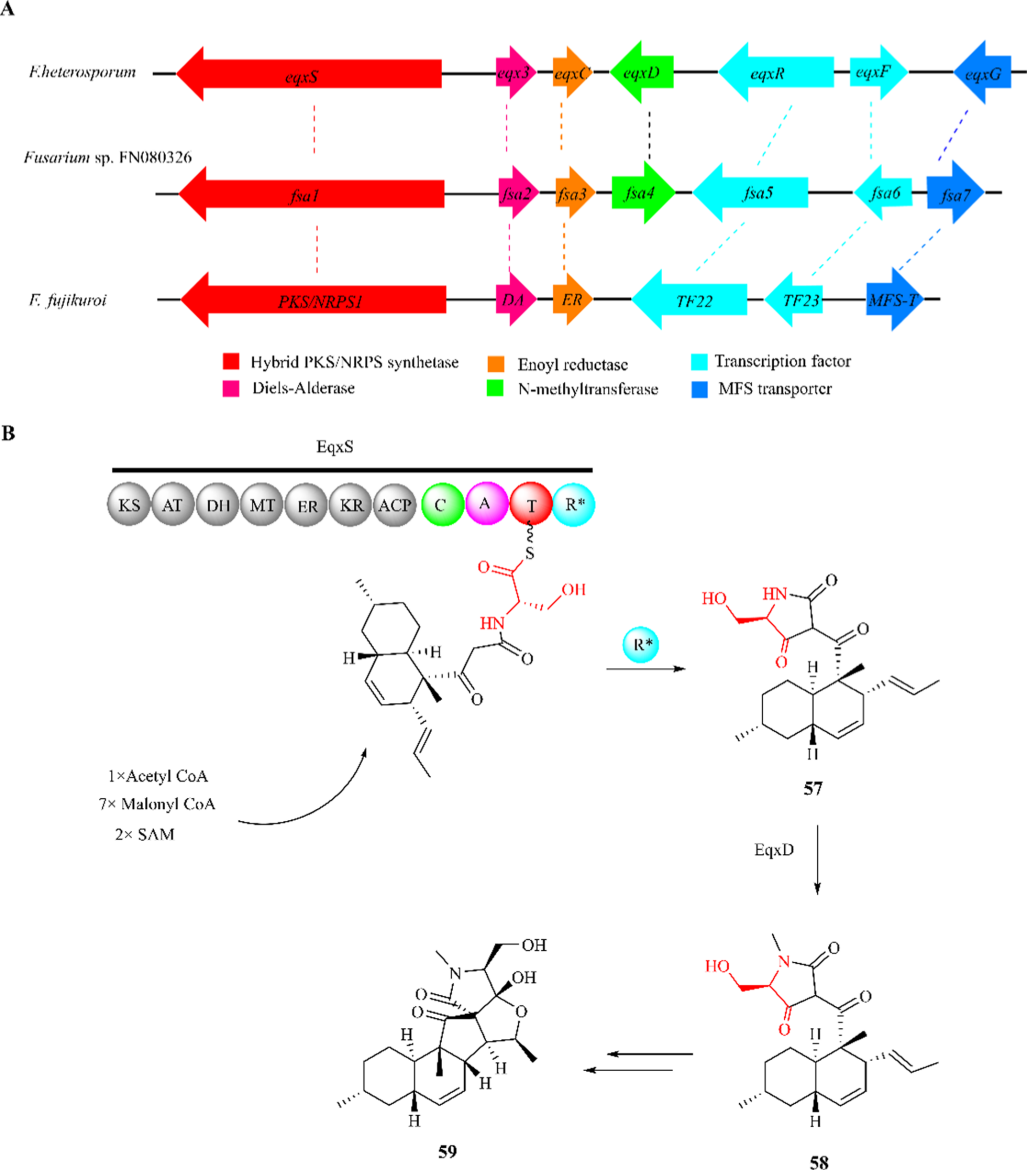
Proposed biosynthesis logic for equisetin (**58**) and fusarisetin A (**59**). **A** The biosynthetic gene cluster related to equisetin biosynthesis in *F. heterosporum, F. fujikuroi* and *Fusarium* sp. FN080326; **B** the proposed biosynthetic pathway of **58** to **59** in *Fusarium* sp. FN080326

Equisetin (**58**) is an HIV-I integrase inhibitor isolated from strain *F. equiseti* NRRL 5537 [157, 158]. Compound **58** and its N-desmethyl derivative trichosetin (**57**) represent tetramic acids, which are also widely present in several *Fusarium* species, including *F. heterosporum*, *F. fujikuroi*, and *Fusarium* sp. FN080326 [150, 159]. These compounds exhibit a broad spectrum of biological activities, including antibacterial, antiviral, antifungal, phytotoxic, and cytotoxic effects [158–163]. Gene deletion and overexpression experiments revealed that the trichosetin biosynthesis gene cluster in *F. fujikuroi* did not contain N-methyltransferase (EqxD), resulting in the isolation of the terminal product **57** [151, 162]. The comparison of gene functions for the biosynthesis of equisetin and its derivatives in *F. heterosporum, F. fujikuroi* and *Fusarium* sp. FN080326 is presented in Fig. 15A and Table 1.

**Table 1:** The comparison of gene functions for the biosynthesis of equisetin

*F.heterosporum* ATCC 74349	*F.fujikuroi* IMI58289	*Fusarium* sp. FN080326	Gene functions
*eqxS*	*PKS/NRPS1*	*fsa1*	PKS/NRPS synthetase
*eqx3*	*DA*	*fsa2*	Diels-Alderase
*eqxC*	*ER*	*fsa3*	Enoyl reductase
*eqxD*	*/*	*fsa4*	N-methyltransferase
*eqxR*	*TF22*	*fsa5*	Zn(II)_2_Cys_6_ transcription factor
*eqxF*	*TF23*	*fsa6*	Zn(II)_2_Cys_6_ transcription factor
*eqxG*	*MFS-T*	*fsa7*	Major facilitator superfamily transporter

The proposed biosynthetic scheme for compound **58** and its derivatives involves the utilization of an acetyl-CoA, seven malonyl-CoA, two S-adenosyl-*L*-methionine (SAM) and *L*-serine to form the backbone [164]. The PKS module of EqxS catalyzes with the enoyl reductase (EqxC) to produce a polyketide unit followed by conjugation with a *L*-serine (in red) through the condensation of the NRPS module. The Dieckmann cyclase domain activity (R*) leads to the release of **57**. Compound **57** is then N-methylated by EqxD to form **58**, which was further converted to fusarisetin A (**59**) in *Fusarium* sp. FN080326 (Fig. 15B).

## Conclusions

*Fusarium* is one of excellent producers of NRPS products with a wide range of biological properties. To the best of our knowledge, over 800 SMs produced by *Fusarium* strains have been recorded in the Dictionary of Natural Products (DNP) database and nearly 300 chemicals related to NRPS pathway [165]. This review highlights only fifteen biosynthetic pathways that linked NRPS products with their corresponding BGCs identified in *Fusarium*. Therefore, most of these NRPS compounds linked to their BGCs need to be investigated. More efforts should be made to apply genetic engineering approaches to elucidate the biosynthetic pathways of other *Fusarium* NRPS-encoding compounds and to characterize their key genes and functions.

### Supplementary Information

**Additional file 1. Table S1**. Detail information for NRPS-type secondary metabolites in Fusarium strains and their research methods.

## Data Availability

The datasets generated during and/or analyzed during the current study are available from the corresponding author on reasonable request.

## Abbreviations

NRPS: Non-ribosomal peptide synthetases
BGCs: Biosynthetic gene clusters
SMs: Secondary metabolites
NCBI: National Center for Biotechnology Information
A domain: The adenylation domain
T domain: The thiolation domain
C domain: The condensation domain
CT domain: A subset of the C domain
E domain: The epimerization domain
R domain: The release domain
M: Modules
MFS: Major facilitator superfamily transporter
GAA: Guanidoacetic acid
GABA: γ-Aminobutyric acid
PLP: Pyridoxal-5′-phosphate
AGAT: A process of amidino transfer that requires
*L*-Arg: *L*-Gly aminidotransferase activity
*HO*Gln: *HO*-Glutamine
Cys: Cysteine
LC–MS: Liquid Chromatograph Mass Spectrometer
NMR: Nuclear Magnetic Resonance Spectroscopy
α- KGD: α-Ketoglutaratedependent dioxygenase
ENNs: Enniatins
KIVR: A novel NADPH-dependent 2-ketoisovalerate reductase
*D*-Hiv: *D*-Hydroxy-isovaleryl
SAM: S-adenosylmethionine
BEA: Beauvericin
HICA: α-Hydroxyisocaproic acid
ATMT: The *Agrobacterium*-mediated transformation approach
APF: Apicidin F
TFs: The transcription factor
*L*-pip: *L*-Piperidinic acid
TDO: Tryptophan dioxygenase
DMAT: Dimethylallyl diphosphate transferase
PKS: Polyketide synthases
KS: *β*-Keto synthase
AT: Acyltransferase
KR: *β*-Keto reductase
DH: Dehydrogenase
MT: Methyltransferase
ER or R: Reductase
ACP: Acyl carrier protein
FA: Fusaric acid
OE: Overexpression
SDR: Short-chain dehydrogenases/reductases
FSH: A serine hydrolase family
FA: Fusaric acid
Leu: Leucine
Pro: Proline
Thr: Threonine
Ala: Alanine
Gln: Glutamine
Tyr: Tyrosine
Val: Valine
Ile: Isoleucine
Phe: Phenylalanine
Arg: Arginine
Ser: Serine
Lys: Lysine
DNP: The Dictionary of Natural Products
