# Supplementary material for: Non-ribosomal peptide synthetase (NRPS)-encoding products and their biosynthetic logics in Fusarium
Source: Microb Cell Fact. 2024 Mar 27;23:93. doi: 10.1186/s12934-024-02378-1 (PMC10967133; doi:10.1186/s12934-024-02378-1)
Supplement: Supplementary file 1 — Additional file 1. Table S1. Detail information for NRPS-type secondary metabolites in Fusarium strains and their research methods. [file 12934_2024_2378_MOESM1_ESM.docx]

| Product | Key gene | Strain（*Fusarium*） | Plasmid construction | Identification of gene function | Heterologous host | Bioactivity |
| --- | --- | --- | --- | --- | --- | --- |
| fusahexin | *NRPS4* | *F. graminearum* PH-1 | ﹣ | knockout, overexpression | ﹣ | surface hydrophobicity |
| fusaoctaxin A | *NRPS5*+  *NRPS9* | *F. graminearum* PH-1 | pPICZ_B | knockout | *Pichia pastoris* GS115 | as pathogenic agent |
| fusaoctaxin B |  | *F. graminearum* PH-1 | pPICZ_B | heterologous overexpression | *Pichia pastoris* GS115 | as pathogenic agent |
| gramillin A and B | *GRA1* | *F. graminearum* | pRF-HU2 | targeted gene disruption | ﹣ | host-specific virulence factors |
| chrysogine | *ftchyA* | *F. tricinctum* CGMCC 3.4731 | *S. cerevisiae* BJ5464-NpgA | homologous recombination, in vitro incubation | *Aspergillus nidulans* | ﹣ |
| beauvericin | *BEA1* | *F. proliferatum* LF061 | pRF-HU2 | targeted gene disruption | ﹣ | cytotoxic, apoptotic,  anti-inflammatory, antimicrobial |
| sansalvamide | *NRPS30* | *F. solani* FGSC 9596 | pRF-HU2 | knockout | ﹣ | cytotoxin |
| apicidin F | *APF1* | *F. fujikuroi* IMI58289 | pNDN-OGG | targeted gene disruption | ﹣ | antimalarial |
| fusarochromene | *fscC*  (NRPS-like) | *F. equiseti* | ﹣ | homologues comparison, ^13^C-labelled | ﹣ | ﹣ |
| fusaristatin A | *NRPS7*+*PKS6* | *F. graminearum* | pRF-HU2E | targeted gene deletion | ﹣ | growth-inhibitory activity |
| W493 B | *NRPS32*+  *PKS40* | *F. pseudograminearum* | pRF-HU2E | targeted gene deletion | ﹣ | antibacterial |
| fusaric acid | *FUB1*+*FUB8* | *F. fujikuroi* IMI58289 | YHR [2,3] | knockout, complementation, overexpression | ﹣ | phytotoxicity |
| fusarin C | *fus1* | *F. fujikuroi* IMI58289 | YHR | knockout, overexpression | ﹣ | estrogenic agonist |
| oxysporidinone | *osdE* | *F. oxysporum* ACCC 36465 | YHR | homologous recombination, in vitro incubation | *A. nidulans* A1145  *S. cerevisiae* RC01 | antifungal |
| fusaridione A | *fsdS* | *F. heterosporum* ATCC 74349 | ﹣ | targeted gene disruption | ﹣ | ﹣ |
| equisetin | *eqxS* | *F. heterosporum* ATCC 74349 | *S. cerevisiae* BY4741 | knockout,  overexpression,  in vitro protein expression, purification | ﹣ | antimicrobial |
| trichosetin | *PKS/NRPS1* | *F. fujikuroi* IMI58289 | YHR | targeted gene deletion, overexpression | ﹣ | antimicrobial |
| fusarisetin A | *fsa1* | *Fusarium* sp. FN080326 | Pbi121 [4] | targeted gene deletion | ﹣ | cytotoxic |

**Reference**

1. Malz S, Grell MN, Thrane C, Maier FJ, Rosager P, Felk A, et al. Identification of a gene cluster responsible for the biosynthesis of aurofusarin in the *Fusarium graminearum* species complex. Fungal Genet Biol. 2005;42(5):420-433.
2. Schumacher J. Tools for botrytis cinerea: new expression vectors make the gray mold fungus more accessible to cell biology approaches. Fungal Genet Biol. 2012;49(6):483-497.
3. Wagner JM, Alper HS. Synthetic biology and molecular genetics in non-conventional yeasts: Current tools and future advances. Fungal Genet Biol. 2016;89:126-136.
4. Wang J, Sui X, Ding Y, Fu Y, Feng X, Liu M, et al. A fast and robust iterative genome-editing method based on a Rock-Paper-Scissors strategy. Nucleic Acids Res. 2021;49(2):e12.
5. Nielsen MR, Wollenberg RD, Westphal KR, Sondergaard TE, Wimmer R, Gardiner DM, et al. Heterologous expression of intact biosynthetic gene clusters in *Fusarium graminearum*. Fungal Genet Biol. 2019;132:103248.
6. Zhang X-W, Jia L-J, Zhang Y, Jiang G, Li X, Zhang D, et al. In planta stage-specific fungal gene profiling elucidates the molecular strategies of *Fusarium graminearum* growing inside wheat coleoptiles. Plant Cell. 2012;24(12):5159-76.
7. Sieber CM, Lee W, Wong P, Münsterkötter M, Mewes HW, Schmeitzl C, et al. The *Fusarium graminearum* genome reveals more secondary metabolite gene clusters and hints of horizontal gene transfer. PLoS One. 2014;9(10):e110311.
8. Varga J, Kocsubé S, Tóth B, Mesterházy A. Nonribosomal peptide synthetase genes in the genome of *Fusarium graminearum*, causative agent of wheat head blight. Acta Biol Hung. 2005;56(3-4):375-388.
9. Zhang H, Ruan C, Bai X, Zhang M, Zhu S, Jiang Y. Isolation and identification of the antimicrobial agent beauvericin from the endophytic *Fusarium oxysporum* 5-19 with NMR and ESI-MS/MS. Biomed Res Int. 2016;2016:1084670.
10. Malz S, Grell MN, Thrane C, Maier FJ, Rosager P, et al. Identification of a gene cluster responsible for the biosynthesis of aurofusarin in the *Fusarium graminearum* species complex. Fungal Genet Biol. 2005;42(5):420-433.
11. Jin JM, Lee S, Lee J, Baek SR, Kim JC, Yun SH, et al. Functional characterization and manipulation of the apicidin biosynthetic pathway in *Fusarium semitectum*. Mol Microbiol. 2010;76(2):456-466.
12. Brown DW, Butchko RA, Busman M, Proctor RH. Identification of gene clusters associated with fusaric acid, fusarin, and perithecial pigment production in *Fusarium verticillioides*. Fungal Genet Biol. 2012;49(7):521-532.
